# Evolutionary Prevalence of the Electrostatic Switch Mechanism in P-type ATPases

**DOI:** 10.1007/s00232-026-00377-4

**Published:** 2026-05-26

**Authors:** Nikita S. Badve, Alyssa V. Buda, Jonas A. Káral, Zhitong Li, Taylah L. Percival, Sofia Y. Wardman, Sophia G. Tran, Simon Y. W. Ho, Ronald J. Clarke

**Affiliations:** 1 School of Chemistry, University of Sydney, Sydney, NSW 2006 Australia; 2 School of Life and Environmental Sciences, University of Sydney, Sydney, NSW 2006 Australia; 3 The University of Sydney Nano Institute, Sydney, NSW 2006 Australia

**Keywords:** Sodium pump, Proton pump, Flippase, Regulation, Lipid, Membrane protein

## Abstract

P-type ATPases are active transporter enzymes that maintain ion electrochemical gradients for functions such as muscle contraction, kidney function, digestion, and nerve signalling. The dysfunction of these ATPases has been linked to several neurological, cardiovascular, and metabolic disorders. An electrostatic switch mechanism (ESM) has been hypothesised to be involved in the regulation of the sodium-potassium pump (Na^+^,K^+^-ATPase) and the gastric proton pump (H^+^,K^+^-ATPase). An analysis of primary and tertiary protein structures suggests a potentially higher prevalence of the ESM in human P-type ATPases than previously thought. Evidence for the ESM was found on the N-terminus for the P1B, and P2C subfamilies of P-type ATPases, and on the C-terminus for the P2A and P5 subfamilies. In the case of the P4 family, evidence for the ESM was found on the N-terminus for some subfamilies and on the C-terminus for others. Evidence for the ESM has been identified in P-type ATPase subfamilies distributed in separate lineages of the phylogenetic tree, suggesting that the ESM evolved multiple times independently.

**Graphical Abstract:**

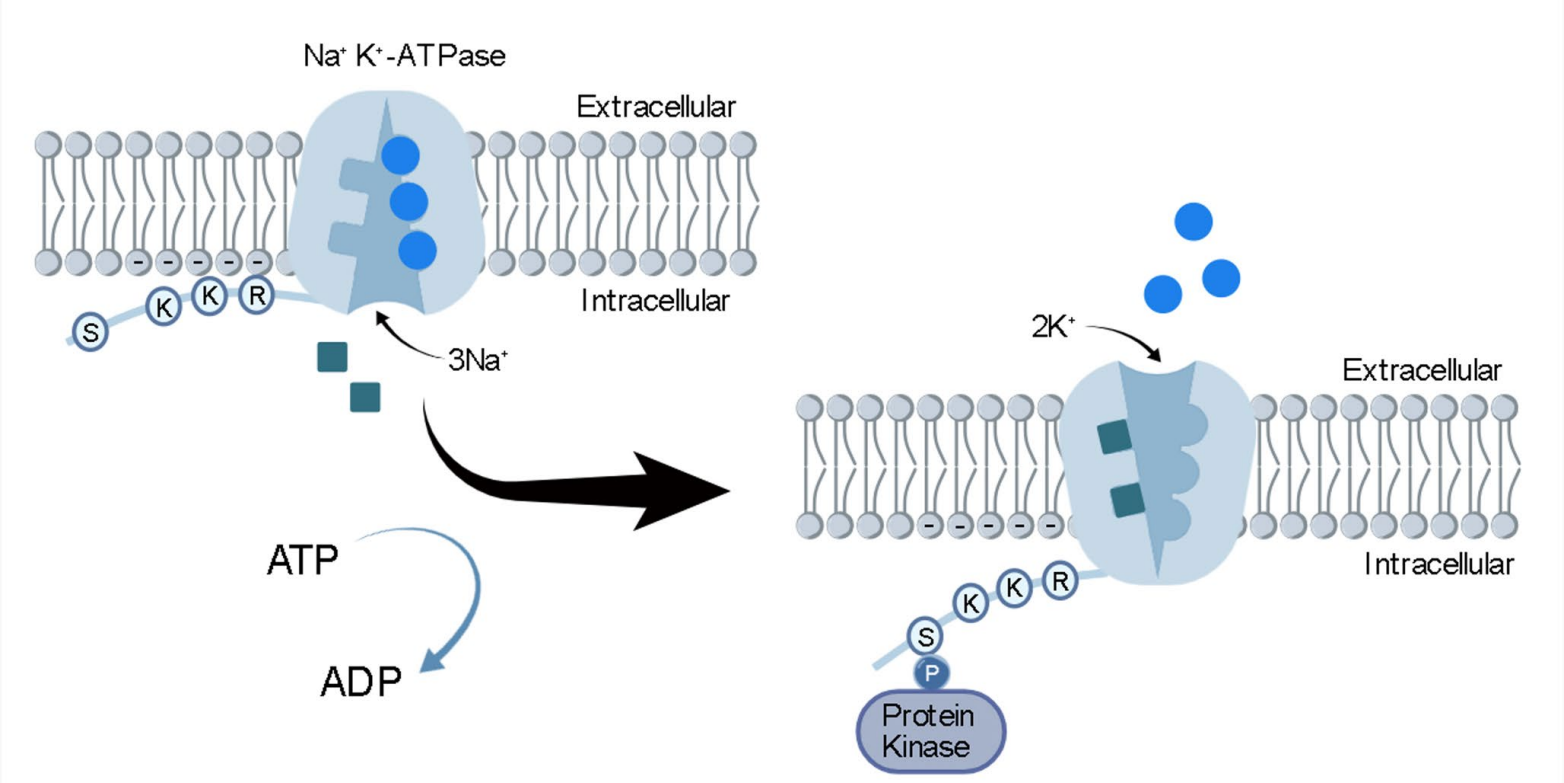

**Supplementary Information:**

The online version contains supplementary material available at 10.1007/s00232-026-00377-4.

## Introduction

The electrostatic switch mechanism (ESM) is a regulatory mechanism of membrane proteins. It was first proposed by McLaughlin and Aderem (1995) as a mechanism whereby the trafficking of peripheral membrane proteins is controlled. Evidence supporting control of trafficking by the ESM has since been presented for numerous peripheral membrane proteins (Seykora et al. 1996; Magee and Marshall 1999; Taniguchi 1999; Murray et al. 2002; Yeung et al. 2008;. Maures et al. 2011; Li et al. 2013; Noguera-Salvà et al. 2017; Clarke 2023). These proteins are held at the cytoplasmic face of the plasma membrane via two forces. Firstly, the proteins have a post-translational modification to either their N- or C-terminus in which a hydrocarbon chain is added, often a myristoyl chain. This inserts itself into the adjacent membrane and acts as a hydrophobic anchor. Secondly, the N- or C-termini of these proteins have positively charged polybasic clusters, usually lysine, which interact attractively with the negatively charged cytoplasmic face of the plasma membrane. The negative charge of the membrane is due predominantly to the negatively charged headgroups of phosphatidylserine, which is concentrated on the cytoplasmic side of the membrane by the action of lipid flippases (Bretscher 1972; Op den Kamp 1979; Zachowski and Devaux 1990; Devaux 1992; Zachowski 1993; Leventis and Grinstein 2010; Clarke et al. 2020). The proteins are thus held at the cytoplasmic surface of the membrane by a combination of both hydrophobic and electrostatic forces, but neither alone is sufficient to hold the protein on the membrane. If one of the forces is broken, the protein is released from the membrane and can move elsewhere within the cell. This is where the ESM comes into play. Apart from basic residues on their N- or C-termini, the proteins also have serine, tyrosine, and/or threonine residues, which can be phosphorylated by protein kinases. This adds negative charge to the N- or C-terminus, at least partially neutralising the positive charges of the polybasic residues and weakening the electrostatic attraction sufficiently that the protein can be released from the membrane.

The hallmarks of the ESM can thus be summarised by four features:

1. An N- or C-terminus protruding into the cytoplasm able to interact with the cytoplasmic membrane surface.
2. Polybasic clusters (positively charged lysine or arginine residues) located on the protein’s N- or C-terminus.
3. Serine, tyrosine or threonine residues located near the polybasic clusters serving as phosphorylation sites of protein kinases.
4. A negatively charged cytoplasmic membrane surface (which is also present in all subcellular organelle membranes except the endoplasmic reticulum).

All of these hallmarks have been found in the Na^+^,K^+^-ATPase and the gastric H^+^,K^+^-ATPase (Clarke 2018), and the results of both biophysical experiments and theoretical simulations have been consistent with an interaction of the N-termini of both proteins with the neighbouring cytoplasmic surface of the membrane (Jiang et al. 2017; Nguyen et al. 2018; Hossain et al. 2021; Lev et al. 2023). Of course, this interaction cannot have anything to do with trafficking of the proteins around the cell, because they are integral membrane proteins, not peripheral membrane proteins, and as such are permanently anchored in the membrane by their transmembrane helices. However, experiments in which the ionic strength was varied to break electrostatic interactions in regions of the Na^+^,K^+^-ATPase structure exposed to the aqueous medium showed that breaking the interactions caused a shift in protein conformation from the E2 state to the E1 state (Jiang et al. 2017). This suggests that in these P-type ATPases, the ESM could play a regulatory role, changing the distribution between E1 and E2 states and thereby affecting the proteins’ ion pumping turnovers.

If the ESM is operative in the Na^+^,K^+^- and gastric H^+^,K^+^-ATPases, it might also play a role in other members of the P-type ATPase family. The purpose of this paper is to provide an overview of P-type ATPases in modern humans (*Homo sapiens*), focussing on the identification of ATPases that bear the four hallmarks of the ESM listed above.

## Results

### Primary Sequence Analysis

Beyond the proposed Na^+^,K^+^-ATPase and gastric H^+^,K^+^-ATPase candidates for the ESM, a total of 13 additional P-type ATPases present in humans were identified to contain a polybasic cluster and a potential phosphorylation site at either their N- or C- termini. These additional P-type ATPases include isoforms (i.e., versions of an ATPase with slightly different amino acid sequences which can be expressed in different tissues), but they do not include splice variants, i.e., shorter versions of an ATPase created by the splicing together of different exons of the protein’s gene. The additional P-type ATPases identified are all so-called canonical forms.

Structural modelling using DeepTMHMM, a deep learning model for transmembrane topology prediction and classification, confirmed that all of the polybasic regions were located within cytoplasmic domains, rather than being part of a transmembrane helix. The N-terminal and C-terminal tails are here defined as the total lengths (in amino acid residues) of the N- and C-terminus, respectively, that lie outside the membrane in the adjacent cell cytoplasm. The lengths of the N- and C-terminal tails were also determined using DeepTMHMM.

The location of the N- or C-terminal tails within the cytoplasm allows for potential interaction with negatively charged membrane surfaces. Polybasic clusters were identified in both the N- and C-termini of several human P-type ATPases, although their distribution varied across families and isoforms (see Table 1).

**Fig. 1:**
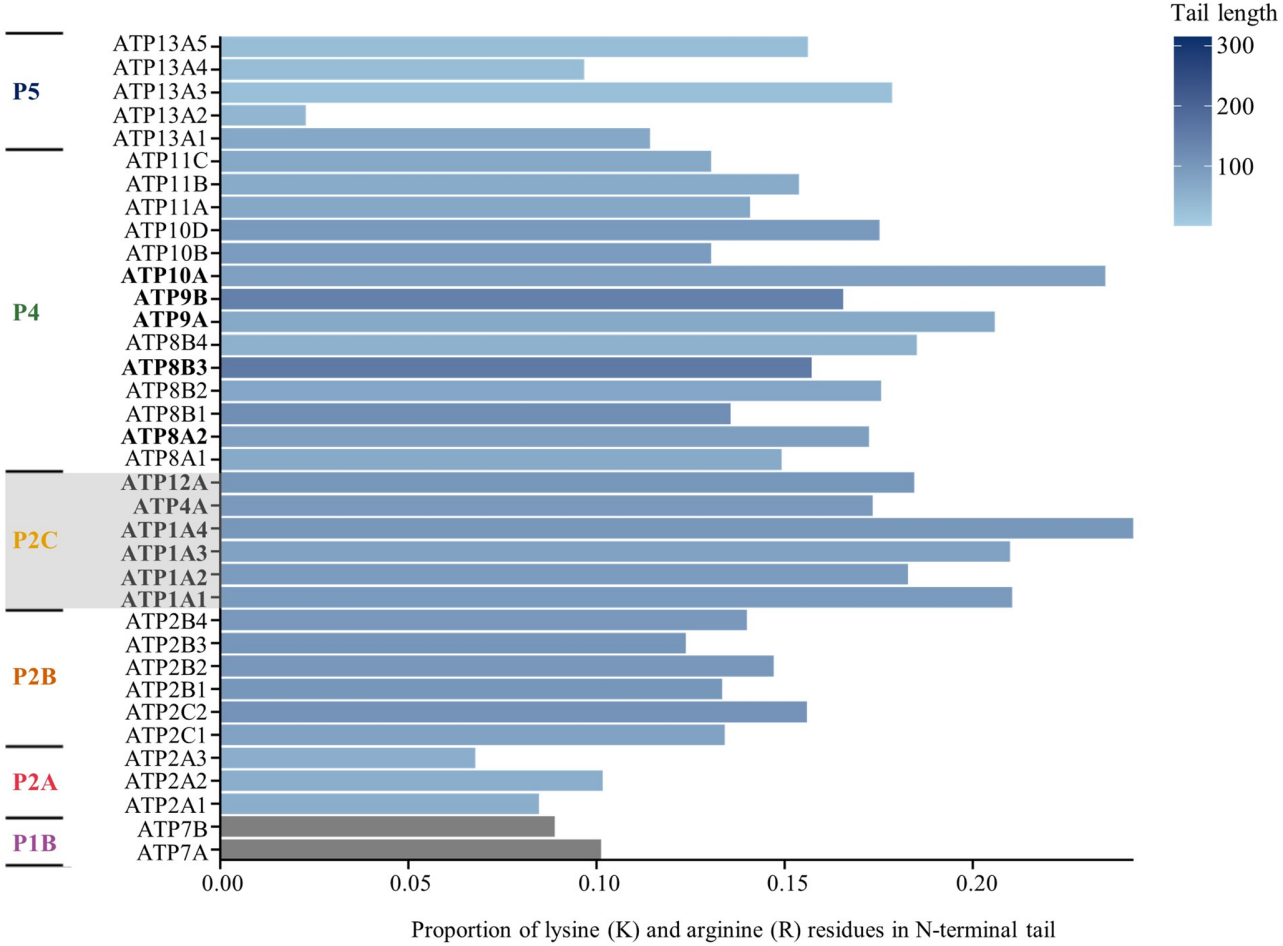
Proportion of of lysine (K) and arginine (R) residues in the N-terminal tails of P-type ATPases. Bar length represents the proportion of lysine (K) and arginine (R) residues relative to tail length, while bar colour indicates tail length. The P1B family has tail lengths of 652 residues and is therefore shown in grey, as this exceeds the upper limit of the colour scale. Terminal tail regions were defined using DeepTMHMM. Proteins in the P2C family have been previously identified as strong candidates for the electrostatic switch mechanism

Candidate clusters on the N-terminus were detected in members of the P4 family, as well as in members of the P2C family which have previously been identified (Clarke 2018; Blayney et al. 2023; Lee et al. 2024). Within the P4 family, ATP8B3, both isoforms of ATP9, ATP10A, and ATP8A2 contained polybasic clusters. The non-gastric H^+^,K^+^-ATPase, ATP12A, of the P2C family was also found to have a polybasic cluster on its N-terminus. No polybasic clusters were found in the P1B, P2A, or P2B families (see Table 1; Fig. 3). Among all N-terminal candidates, P2C ATP1A4, i.e., the alpha4 subunit of the Na^+^,K^+^-ATPase, displayed a lysine plus arginine (K + R) residue density of 0.243, the highest of all P-type ATPases in which terminal polybasic clusters were identified (Fig. 1).

**Fig. 2:**
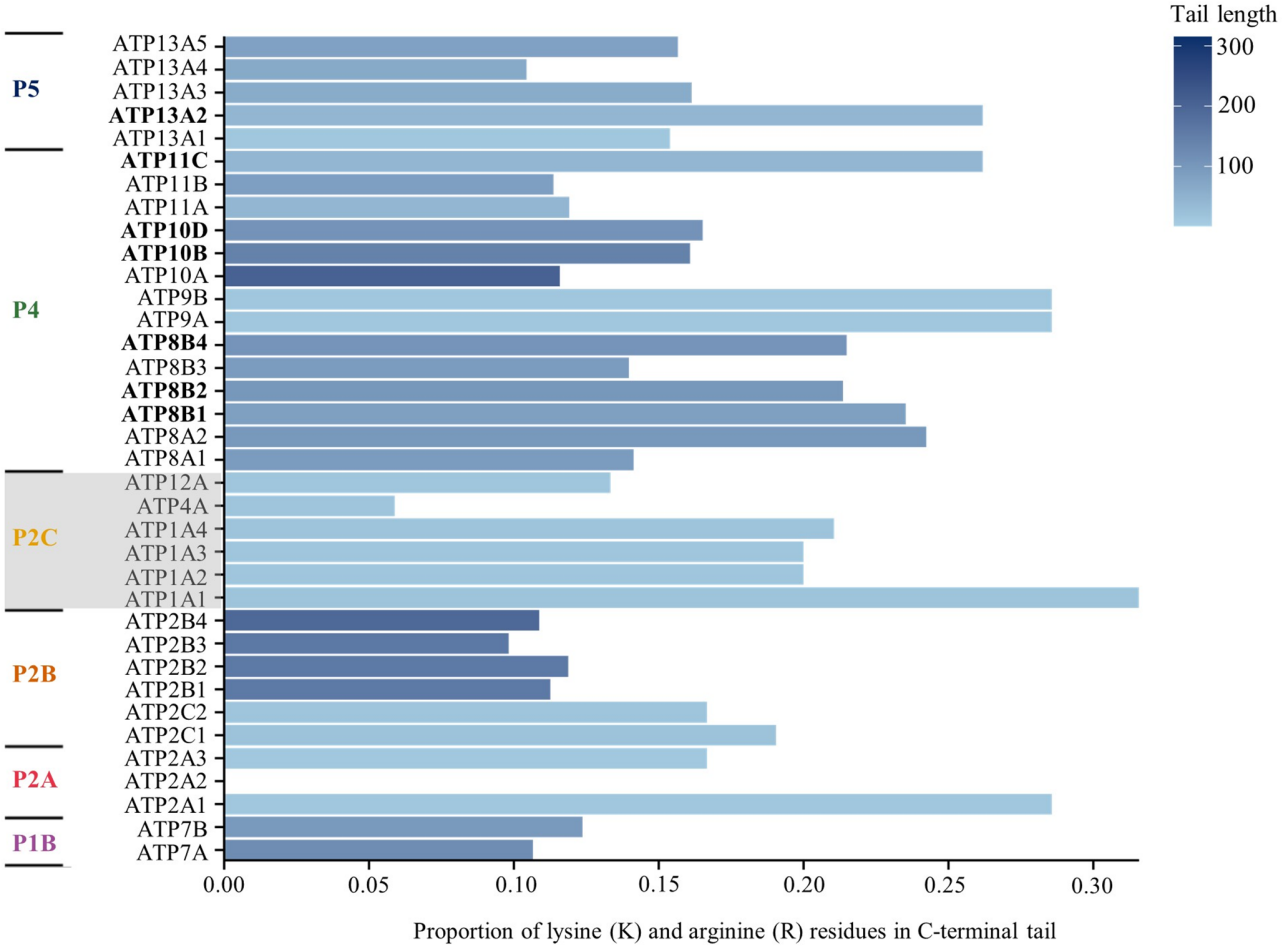
Proportion of lysine (K) and arginine (R) residues in the C-terminal tails of P-type ATPases. Bar length represents the proportion of lysine (K) and arginine (R) residues relative to tail length, while bar colour indicates tail length. ATP2A2 contains no lysine or arginine residues in its C-terminal tail

**Fig. 3:**
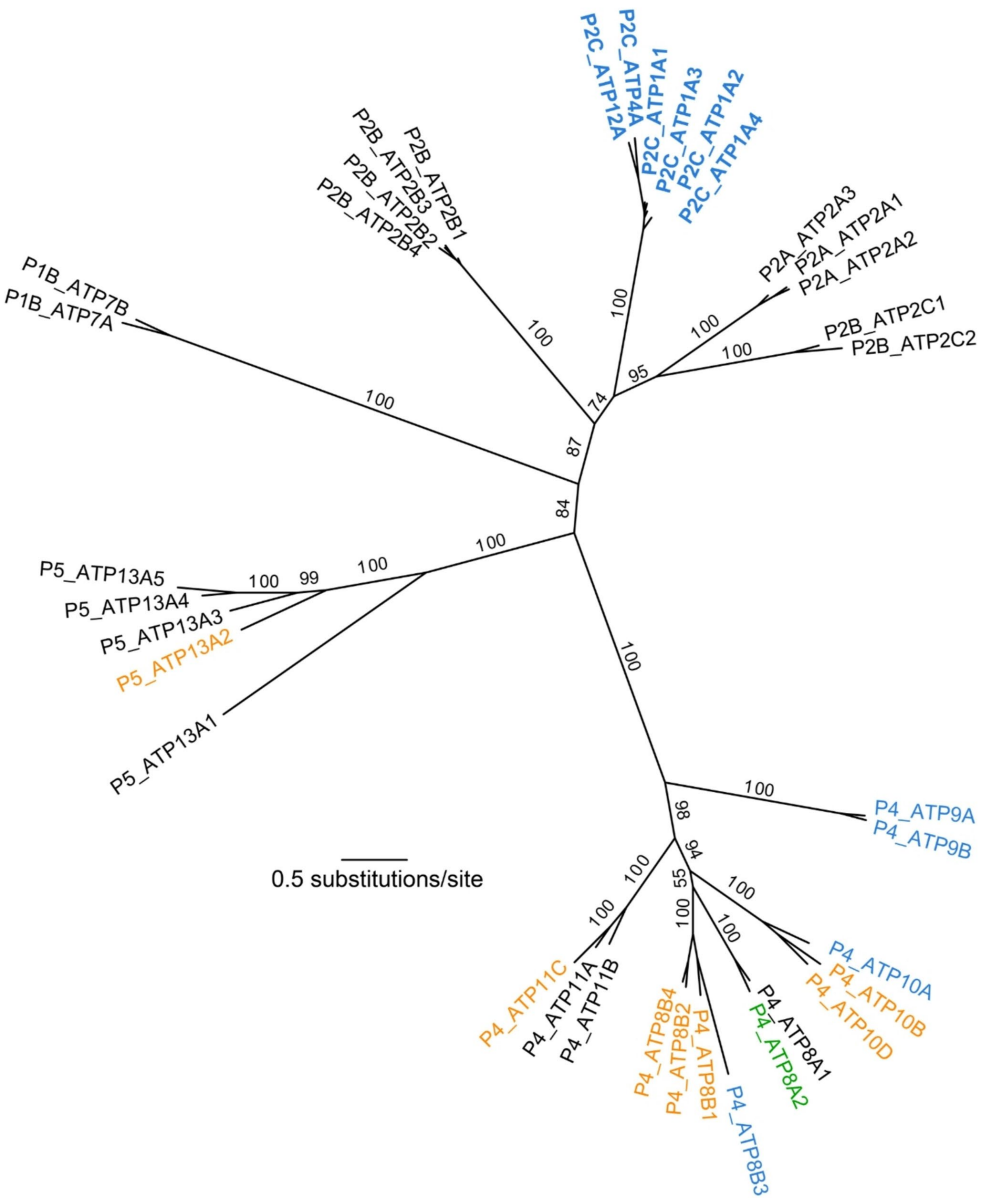
Unrooted phylogenetic tree showing the evolutionary relationships among P-type ATPases in humans, inferred using maximum likelihood. Branches are labelled with bootstrap support values, given as percentages. The scale bar indicates substitutions per site, which is an indication of evolutionary distance. Tip labels in blue font signify proteins with a polybasic cluster at their N-termini. Tip labels in orange font signify proteins with a polybasic cluster at their C-termini. P4 ATP8A2, which is shown in green font, has polybasic clusters at both its N- and its C-termini. The P2C labels are in bold font, to indicate that these were known previously

Candidate polybasic clusters on the C-terminus were identified in proteins belonging to the P4 and P5 families. Within the P5 group, ATP13A contained polybasic clusters in one of its five isoforms. Several P4 ATPases also satisfied the selection criteria. Two isoforms of ATP10, as well as one isoform of ATP8A, all three isoforms of ATP8B, one isoform of ATP11, and one isoform of ATP13A satisfied the selection criteria (Table 1 ; Fig. 3). P2C ATP1A1, i.e., the alpha1 subunit of the Na^+^,K^+^-ATPase, exhibited a K + R density of 0.316, the highest among all C-terminal candidates (Fig. 2). Notably, ATP8A2 within the P4 family of P-type ATPases possessed polybasic clusters on both its N- and C-terminus. The other isoform, ATP8A1, however, did not contain any polybasic clusters on either its N- or C-terminus.

**Table 1:**
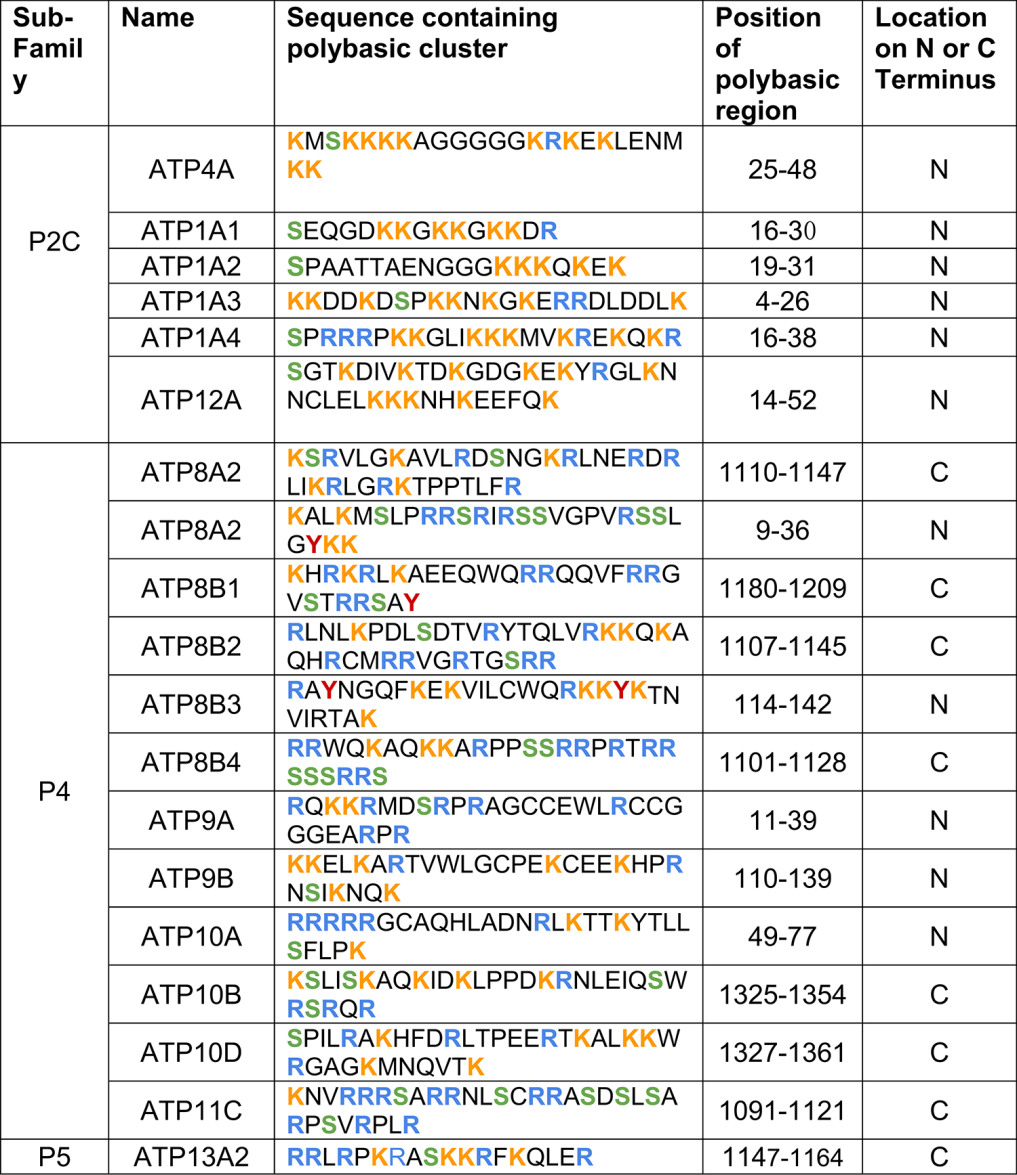
Human P-type ATPases with identified polybasic clusters within their N- or C- termini. Sequences were treated as potential candidates of the electrostatic switch mechanism if they had ≥ 9 basic residues (lysine [K] or arginine [R]) and were located adjacent to a known phosphorylation site (serine [S] or tyrosine [Y]), in a window of 30 amino acids. The amino acid positions of each polybasic region are indicated. All of the polybasic clusters identified are located on the cytoplasmic face of the protein

### Phylogenetic Analysis

Phylogenetic analysis grouped the reference sequences, Na^+^, K^+^-ATPase and gastric H^+^, K^+^-ATPase, as a clade of the P2C subfamily. Although the non-gastric H^+^, K^+^-ATPase has not been suggested to be structurally adapted for regulation via the ESM, the phylogenetic analysis shows that it shares a recent evolutionary ancestor with the gastric H^+^, K^+^-ATPase, clustering closely within the same subfamily (see Fig. 3). The P2C branch appears most closely related to the Ca^2+^-ATPases, of the P2B and P2A subfamilies, which also function in active ion transportation, but specialise in the translocation of Ca^2+^ ions. The proximal positioning of these families has a moderately strong bootstrap support value of 87%.

### Electrostatic Potential Maps

**Fig. 4:**
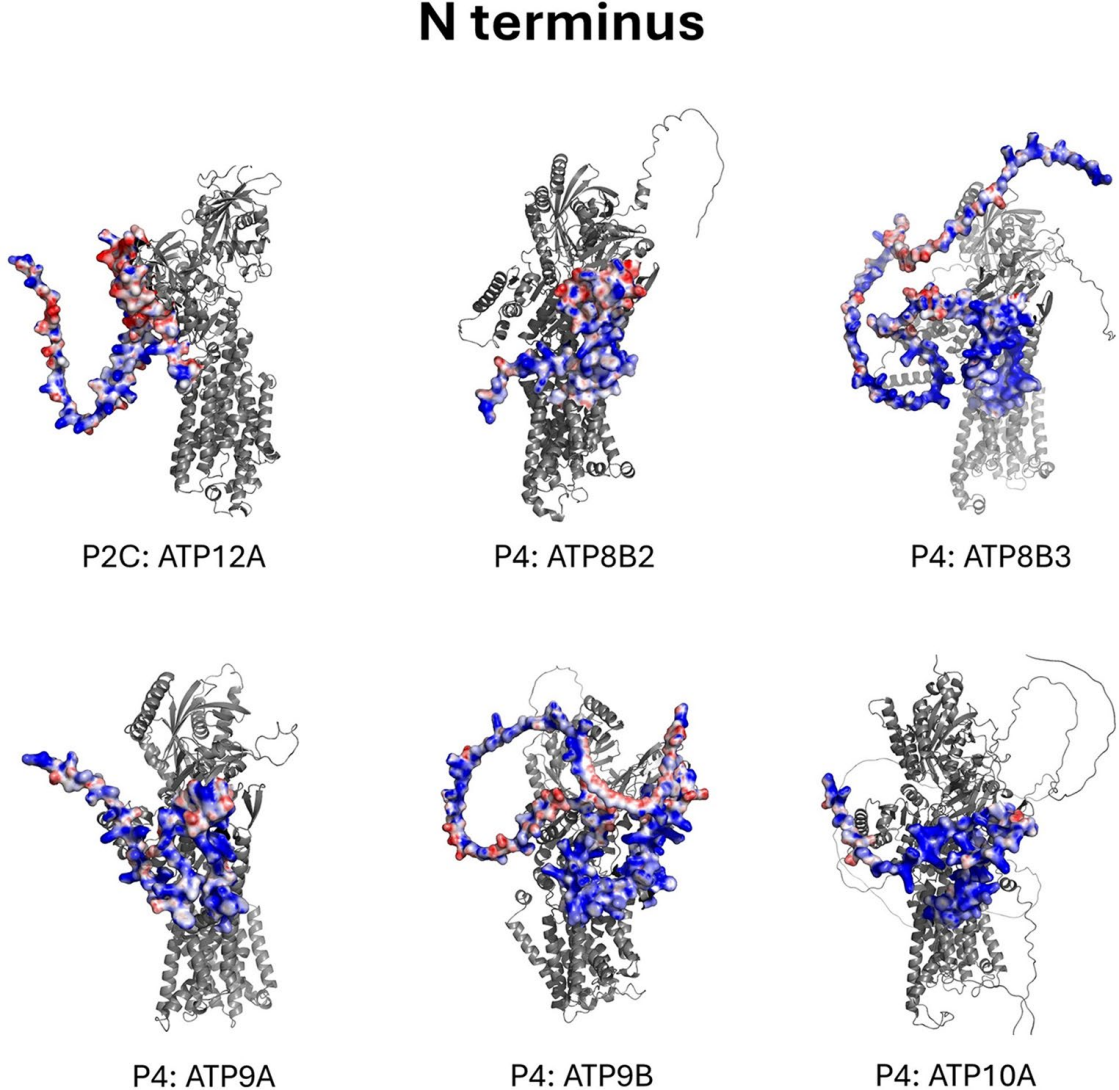

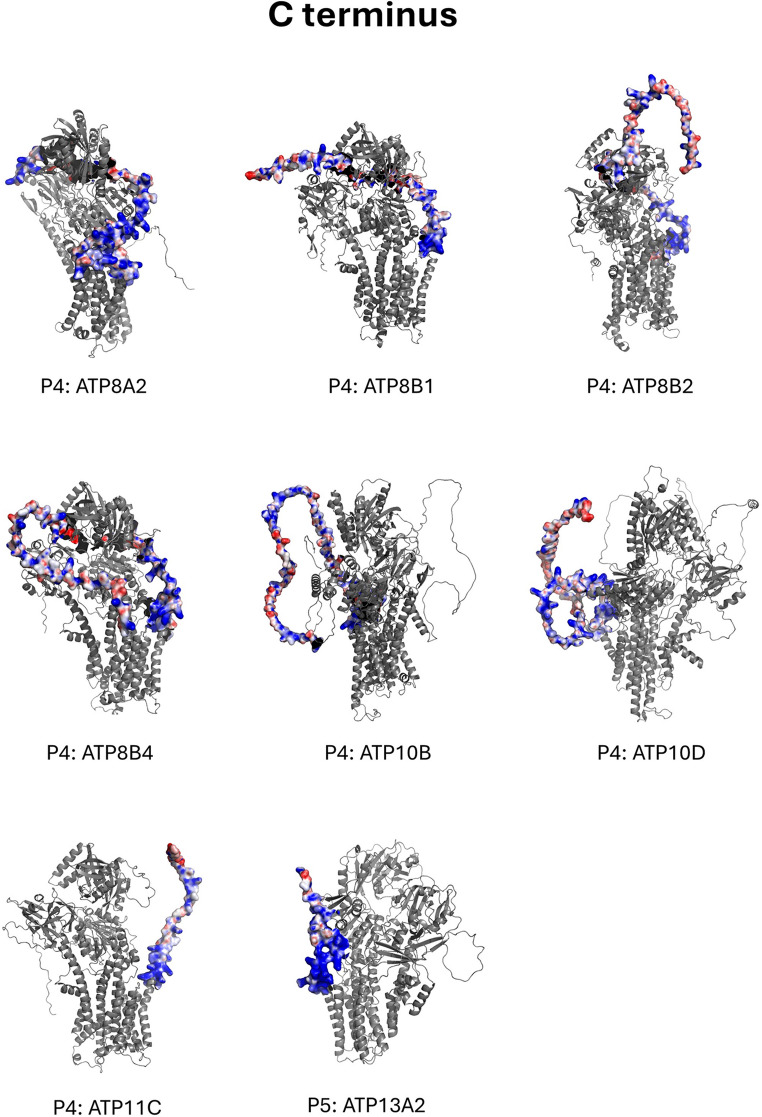
Tertiary structure analysis of human P-type ATPases. The surface electrostatic potentials were mapped onto the tertiary sequences using the Adaptive Poisson-Boltzmann Solver and visualised in PyMOL with default parameters. The structures have been grouped according to their P-type ATPase subfamily. Blue regions indicate areas of positive electrical surface potential, *V*, defined as regions where *V* is > 0 and ≤ + 10 *kT*/*e* = 0.26 V. Here *k* is Boltzmann’s constant (1.38⋅10^− 23^ J K^− 1^), *T* is the absolute temperature in K, and *e* is the electronic charge (1.6 × 10^− 19^ C). The red regions represent areas of negative surface potential, defined as regions where *V* is ˂ 0 and ≥ -10 *kT*/*e* = -0.26 V. White constitutes regions with zero electrostatic surface potential. Each structure was visually assessed for the presence of a polybasic, positive region (as depicted by deep blue colouring) on the N- or C- terminal tails. Colour depth indicates the magnitude of the electrostatic surface potential, e.g., a deeper blue indicates a more positive surface potential, whereas a deeper red indicates a more negative surface potential. It is important to note that the N- and C-termini are highly disordered, which is evident from the low confidence ratings of the predicted tertiary structures of these regions on AlphaFold (Jumper et al. 2021). Therefore, the tertiary structures shown should be considered as a snapshot of the N- or C-terminus conformation rather than a static structure

Mapping of the electrostatic surface potential onto the tertiary structures of each P-type ATPase indicated strong positive charges on the N-terminal ends of the Na^+^,K^+^-ATPase (ATP1A1, ATP1A2, ATP1A3, ATP1A4), the gastric (P2C ATP4A) and non-gastric (P2C ATP12A) H^+^,K^+^-ATPase, and two isoforms of the phospholipid flippase P4 ATP9, which is found in the membrane of the Golgi apparatus. Strong positive charges were also found on three other phospholipid flippases belonging to the P4 family: ATP8A2, ATP8A2, and ATP8B3. Further, several other proteins displayed strong positive charges on their C-terminal ends: three isoforms of the P4 lipid flippase ATP8B, two isoforms of the P4 lipid flippase ATP10, which is located in the membranes of late endosomes and lysosomes, the P4 lipid flippase ATP11C, and the P5 ATPase ATP113A2, which transports inorganic cations and polyamines across the lysosomal membrane. These findings are consistent with the primary sequence analysis (Figs. 1 and 2), which confirmed the presence of positively charged residues capable of generating these electrostatic features and suggests that they remain exposed rather than being shielded during folding (Fig. 4).

It is worth mentioning here that electrostatic surface potential has recently been suggested as one of six fundamental physicochemical parameters that could be used to define life itself (Fantini et al. 2024), the others being time, water, entropy, space, and quantum mechanisms. The regulation of P-type ATPases via an electrostatic switch mechanism, which fundamentally relies on electrostatic surface potentials, supports the view that electrostatic surface potential is of primary importance in membrane biology and is possibly a parameter that underpins the biology of living systems.

Concerning our predictions of protein tertiary structures (Fig. 4), it is important to note that AlphaFold does not take into account the influence of the lipid bilayer environment on protein conformation. In principle it would be possible to insert these structures into a biological membrane theoretically using the Positioning Proteins in Membranes (PPM) server, as has been done in an accompanying article in this issue (Fairuz et al. 2026). This would allow one to judge whether the N- or C-terminal tails could reach the membrane surface, but it would still not account for any changes in protein conformation due to interaction with the lipid bilayer. This is a further reason, in addition to the intrinsic disorder of the N- and C-terminal tails, why our predictions of protein tertiary structures should be interpreted cautiously.

### Transverse Lipid Asymmetry of the Membrane

To determine whether the surrounding membrane environment could support electrostatic interactions required for the electrostatic switch mechanism, a literature review of membrane lipid asymmetry was conducted. The analysis indicated that all candidate ATPases are localised in asymmetric membranes, which would facilitate interactions between negatively charged phospholipid headgroups, such as that of phosphatidylserine, and polybasic clusters on the N- or C-terminus of the ATPase.

Several candidates are located in the plasma membrane, where the electrostatic switch mechanism has previously been described (Clarke 2018; Blayney et al. 2023; Lee et al. 2024). These include all isoforms of the Na^+^,K^+^-ATPase (ATP1A1–ATP1A4), both the gastric and the non-gastric H^+^,K^+^-ATPase (ATP4A and ATP12A), and the members of the P4 family ATP10A, ATP10D, ATP8A2, ATP8B1, ATP8B2, ATP8B4, and ATP11C. Several of the candidate ATPases are located in the membranes of subcellular organelles with transverse lipid asymmetry. These include members of the P4 family ATP9A, ATP9B, ATP10B, and ATP8B3, and the P5 family member ATP13A2. Of the ATPases in subcellular organelles, the P4 members ATP9A, ATP9B, and ATP10B are all thought to be phospholipid flippases. Via flippase activity, they would, therefore, create transverse lipid asymmetry in the membrane in which they are located. The P5 ATPase ATP13A1 is a polyamine transporter and is located within the membrane of lysosomes and late endosomes, which also possess highly asymmetric membranes. It is worth noting that no candidate P-type ATPase is located in the membrane of the endoplasmic reticulum, which has a symmetric membrane, due to the activity of scramblases.

For potential regulation via an electrostatic switch mechanism, lipids with anionic headgroups must be concentrated in the membrane leaflet in which a polybasic terminal tail of the P-type ATPase is located. Data on the lipid asymmetries of each membrane were derived from experimental studies and reviews (Zachowski 1993; Kurz et al. 2011; Van Meer et al. 2008; Cai et al. 2009; Van Meer 2011; Hankins et al. 2014; Ebner et al. 2025; Kaur et al. 2025).

## Discussion

The aim of this study was to identify the P-type ATPases most likely to be regulated by an electrostatic switch mechanism and to infer the evolutionary relationships among these proteins. The results support the conclusion that the electrostatic switch mechanism is widespread among P-type ATPases. In addition to the Na^+^,K^+^-ATPase and the gastric H^+^,K^+^-ATPase, which have been investigated previously (Clarke 2018), the analyses reported here indicate that several phospholipid flippases are also likely to be regulated by an electrostatic switch mechanism.

Six P-type ATPases containing a polybasic cluster and a potential phosphorylation site in close proximity within the N-terminus were identified as possible candidates from primary analysis for regulation by the electrostatic switch mechanism (ESM). This is in addition to the four isoforms of the alpha subunit of the Na^+^,K^+^-ATPase and the gastric H^+^,K^+^-ATPase. Within the P4 family, ATP8B3 and both isoforms of ATP9 (A and B) satisfied these criteria. The preservation of these positively charged regions across both ATP9 isoforms suggests functional conservation, supporting the possibility that they contribute to a shared regulatory mechanism.

Among these proteins, ATP9A is a phospholipid flippase localised in the Golgi apparatus and endosomes, membranes that maintain lipid asymmetry. Its N-terminus contains nine basic residues in the first 40 amino acid residues and a nearby serine residue (Table 1). Consistent with these features, our analysis shows a lysine + arginine (K + R) density of 0.206 in this region (Fig. 1), indicating an enrichment of positively charged residues. Phosphorylation of the serine could alter the local charge environment and modulate interactions between the N-terminus and the membrane.

Similar features were identified in ATP9B, another Golgi-localised phospholipid flippase. Its N-terminus shows a K + R density of 0.166 with adjacent serine and tyrosine residues (Fig. 1; Table 1). ATP8B3, another P4 phospholipid flippase, has been reported to localise to the acrosome membrane in sperm cells. Notably, the acrosomal membrane is enriched in negatively charged lipids on the luminal leaflet (Kurz et al. 2005).

Eight candidate proteins with a polybasic cluster on the C-terminus were identified from primary analysis, and all proteins were located on a membrane exhibiting lipid asymmetry (Fig. 1; Table 1). The Golgi-associated flippase ATP8B4 exhibits a strong polybasic cluster with a K + R density of 0.215 and several nearby serine residues (Zachowski 1993; van Meer et al. 2008; Shin and Takatsu ).

ATP10D in the P4 family shows a K + R density of 0.165 in its C-terminal region (Fig. 1) and a serine residue close to the polybasic cluster (Table 1). However, Okamoto et al. (2020) reported that swapping the N- or C-termini did not alter the flippase activity of ATP10D, suggesting that the C-terminus does not play a major regulatory role in enzymatic activity. Therefore, it is doubtful that ATP10D is regulated by an ESM.

Other members of the P4 family found to contain a polybasic cluster at the C-terminus include ATP8A2, ATP8B1, ATP8B2, ATP10B, and ATP11C (Fig. 2; Table 1). In the P5 family, ATP13A2 was also found to contain a polybasic cluster at the C-terminus. However, the fact that other isoforms of ATP13A do not exhibit polybasic clusters (Fig. 3), casts doubt on regulation of this enzyme by an ESM, because, if this were the case, one might expect regulatory regions to be conserved across isoforms.

The aminophospholipid flippase, ATP11C, was found to have a polybasic cluster, predominantly consisting of arginine residues, on its C-terminus. The ATP11C protein is found in the plasma and endosome membranes, which exhibit lipid asymmetry (Zachowski 1993; van Meer et al. 2008; Clarke et al. 2020). There are several serine residues that could potentially act as sites of phosphorylation. Ser1103 is even registered as a phosphorylation site in the public phosphosite database PhosphoSitePlus (Hornbeck et al. 2004). A study by Takatsu et al. (2017) exchanged the C-termini of ATP11A and ATP11C and found that the ATP11C chimaera with the ATP11A C-terminus was dysfunctional. It can be concluded that there is a regulatory mechanism on the C-terminus specific to ATP11C and not observed in ATP11A, making it a possible candidate for the ESM. However, like ATP13A2, other isoforms of ATP11 do not exhibit polybasic clusters (Fig. 3). Therefore, the case for regulation of this enzyme by an ESM is not entirely convincing.

It also appears doubtful that phosphatidylserine flippase ATP8A2 is regulated by an ESM. Chalat et al. (2017) performed a series of truncation and substitution mutations which established that ATP8A2 is autoinhibited by phosphorylation of Ser1138 within a calcium/calmodulin-dependent protein kinase II target motif (CaMKII). Since the C-terminus is already involved in this regulatory mechanism, it is perhaps unlikely that an ESM operates in a region in such close proximity to another phosphorylation-dependent regulatory sequence.

Another member of the P4 family, ATP8B1, has promising structural features including a polybasic cluster and a corresponding serine (Fig. 1; Table 1), located on the asymmetric apical canalicular membrane (Dawson 2010). However, ATP8B1 is autoinhibited by its N- and C-terminal tails, which form extensive interactions with the catalytic sites (Dieudonne et al. 2022). Ser1223 on the C-terminus was shown through substitution experiments to be subject to regulation via phosphorylation by CaMKII. Furthermore, Arg1128, which was identified as part of the polybasic cluster, was found to be involved in salt bridge interactions with the A- and P-domains of the alpha subunit (Dieudonne et al. 2022). Phosphoinositides are also regulators of ATP8B1 activity, which interact with basic residues including lysine and arginine (Dieudonne et al. 2023). Thus, in this case the polybasic motif identified in the primary sequence analysis appears to serve a functional purpose unrelated to an ESM.

Binding of the N-terminus, the C-terminus, or both within clefts between the N-, P- and A-domains has also been reported for other P-type ATPases (Timcenko et al. 2019; Bai et al. 2019; Li et al. 2021; Cheng et al. 2022). This does not, however, rule out N- or C-terminal interaction with the surrounding membrane, as proposed in the electrostatic switch mechanism. Indeed, analyses of tryptic digestion patterns of the Na^+^,K^+^-ATPase have shown that the location of its N-terminus is conformation dependent, with significant movement occurring on transition between the E2 and E1 conformations (Jørgensen 1975; Jørgensen et al. 1982; Jørgensen and Collins 1986; Jørgensen and Andersen 1988). It is, therefore, possible that the N- and C-termini move during the Albers-Post cycle between sites on the cytoplasmic domains of the protein and sites on the surrounding membrane.

Overall, three P4 ATPases—ATP8B4, ATP9A, and ATP9B—display features consistent with regulation by an ESM and have no reported conflicting regulatory mechanisms. In addition, their terminal regions face the cytoplasmic side of the membrane (Okamoto et al. 2020), which is enriched in negatively charged lipids due to membrane asymmetry. Taken together, these characteristics suggest that these ATPases are the most likely candidates to be regulated by ESM.

An interesting evolutionary pattern emerged, as the members of the P2C family were previously theorised to be regulated by an ESM on their N-terminus (Lee et al. 2024; Blayney et al. 2023), while many of the candidate ATPase proteins of the P4 family identified in this study have polybasic regions on their C-terminus (Table 1). These two subfamilies are not closely related (Fig. 3). This could potentially indicate that an adjacent ESM evolved convergently on the C-terminus. Independent evolution of the same mechanism on two separate sites is not unheard of in P-type ATPases, as calmodulin regulation in Ca^2+^-ATPases is thought to have evolved convergently on the N-terminus in plant cells and C-terminus in animal cells (Palmgren 1998; Palmgren and Axelsen 1998). This scenario of convergent evolution should be further explored to establish why and how the ESM might have evolved twice.

One might wonder why almost no mention has been made here of Ca^2+^-ATPases, which are also prominent members of the P-type ATPase family. The two main Ca^2+^ transporting P-type ATPases are the sarco(endo)plasmic reticulum Ca^2+^-ATPase (SERCA) and the plasma membrane Ca^2+^-ATPase (PMCA). An ESM requires a concentration of negatively charged lipids in one leaflet of the membrane. This is not the case for the endoplasmic reticulum membrane, which is largely symmetric. Thus, one of the prerequisites for an ESM is not fulfilled. The sarcoplasmic reticulum membrane is, in fact, asymmetric, but the structure of SERCA does not depend on what membrane it is embedded in, and therefore, the ESM is not operative here. The situation could be different for the PMCA. The plasma membrane is asymmetric and negatively charged phosphatidylserine is normally concentrated in the internal cytoplasmic leaflet of the membrane, an essential requirement for an ESM. Although PMCA does not possess significant clusters of basic residues on its N- or C-termini, it does have a cluster of basic residues on its first cytoplasmic loop (Bruce 2018), which has been postulated to play a role in its regulation. Because it is covalently bound to transmembrane helices at both ends, the first cytoplasmic loop would have less freedom of movement than the N- or C-terminus, but it could nevertheless be involved in a form of ESM.

One limitation of this study is that only the first and last 100 amino acids of P-type ATPases were searched for polybasic clusters. There are, however, members of the P-type ATPase family that have longer N- or C-termini. This is particularly the case for heavy metal transporters, such as the copper transporter ATP7B, which has six metal-binding domains on its N-terminus (Ariöz et al. 2017). These are required because of the low solubility of copper salts. Heavy metal ATPases have, thus, also been excluded in the present study. Therefore, it is possible that other potential ESM candidates could still be discovered.

### Methods

#### Primary Sequence Analysis

Catalytic subunits of P-type ATPases were identified via the IUPHAR/BPS Guide to PHARMACOLOGY database (Alexander et al. 2019), and sequences were obtained from UniProt (Bateman et al. 2025). The sequences for each protein were selected by UniProt based on their demonstrated functionality, broad expression, orthologous sequence conservation or alignment with genome-curated consensus sequences. In the absence of such information, the longest sequence was selected.

ATPases were screened for polybasic clusters within the N- and C-termini using RStudio (Posit Team 2025) with the Biostrings (Pagès et al. 2025) and stringr (Wickham 2023) packages in R (R Core Team 2023). DeepTMHMM (Hallgren et al. 2022) was utilised to define the lengths and amino acid content of the N- and C-terminal tails, either prior to the first transmembrane passage or following the last, respectively. In DeepTMHMM, neural networks were employed to improve topology predictions of transmembrane alpha-helical proteins, including P-type ATPases.

A sliding-window approach was applied to identify regions containing ≥ 9 lysine (K) or arginine (R) residues within a 30-amino-acid sequence of the defined tails with shorter sequences excluded. Potential phosphorylation sites were assessed by identifying serine (S), or threonine (T) or tyrosine (Y) residues located within the polybasic window or within five residues flanking the window. These parameters balanced sensitivity by capturing potential polybasic clusters and specificity by maintaining a high local positive charge, thereby reducing false positives. This approach ensured the identification of functionally relevant sites, similar to previous studies (Requião et al. 2016). Subsequent analysis calculated the proportion of lysine and arginine residues relative to tail length using the Biostrings (Pagès et al. 2025), ggplot2 (Wickham 2016), and dplyr (Wickham 2023) packages. The full code used for the screening and calculation of N- and C-terminal enrichment is provided in the Supplementary Material.

#### Phylogenetic Analysis

A phylogenetic tree of human P-type ATPases was used to determine the evolutionary relationships of the newly identified ESM candidate proteins to the sodium-potassium (Na^+^, K^+^-ATPase) and gastric proton (H^+^,K^+^-ATPase) pumps, which were both previously hypothesised to contain the ESM (Clarke 2023).

Multiple sequence alignment was performed using the MUSCLE algorithm (Edgar 2004) in MEGA12 (Kumar et al. 2024). After excluding highly divergent sequences that lacked any apparent homology to the majority of the sequences, the remaining sequences were realigned. The resulting sequence alignment was trimmed using trimAl (Capella-Gutiérrez et al. 2009) to remove any sites with > 25% gaps, in order to exclude poorly aligned regions and to retain the more phylogenetically informative conserved regions (Axelsen and Palmgren 1998). The evolutionary relationships among the sequences were inferred using a maximum-likelihood phylogenetic analysis in IQ-TREE 2 (Bui et al. 2020). The best-fitting model of amino acid substitution was selected using the Bayesian information criterion. Support for nodes in the tree was estimated using ultrafast bootstrapping with 1000 replicates.

#### Tertiary Structure Analysis

Protein structures, predicted by AlphaFold 3 (Abramson et al. 2024), were generated to examine tertiary conformations, accounting for protein folding that may position polybasic residues in close spatial proximity or at a distance. This analysis also accounted for instances of convergent evolution, where similar tertiary structures have arisen despite low sequence identity. AlphaFold models derived from UniProt sequences were visualised in PyMOL (The PyMOL Molecular Graphics System, Version 3.1.6.1 Schrödinger, LLC). Experimentally derived crystal structures were not utilised, because they cannot resolve the disordered terminal tails; these are predicted by AlphaFold models, albeit with lower confidence in these regions. However, in the 14th Critical Assessment of protein Structure Prediction (CASP14), AlphaFold models demonstrated competitive accuracy with experimentally derived structures, achieving a median backbone accuracy of 0.96 Å r.m.s.d._95_ in well-ordered regions (Jumper et al. 2021). Nevertheless, disordered terminal tails should be interpreted with caution.

Electrostatic calculations were performed using the Adapative Poisson-Boltzmann Solver (APBS, version is 3.4.1) with the automatic multigrid focusing method (mg-auto) (Jurrus et al. 2018). Prior to APBS calculations, atomic charges, radii, and protonation states were assigned using the PDB to PQR file conversion server, PDB2PQR (version 3.7.1) (Dolinsky et al. 2007). The PDB structure was processed under aqueous conditions at pH 7.0, applying default PDB2PQR algorithms, including steric clash removal, hydrogen bond network optimization, and pH dependent titration state assignment using the Protein pK_a_ (PROPKA) server (Li et al. 2005). Protonation states were assigned to titratable residues based on PROPKA predictions, restricted by Parameters for Solvation Energy (PARSE) force field compatibility; standard ionization states were retained for residues where specific protonation variants were not parameterized.

Electrostatic potentials were calculated by solving the linearized Poisson–Boltzmann equation, applying a solute (protein) dielectric constant of 2 and a solvent dielectric constant of 78.54, corresponding to water, with temperature set to 298.15 K. The solvent was modeled with an ionic strength of 0 M. Resulting electrostatic potential maps were exported as DX files and mapped onto AlphaFold-derived protein structures for visualization in PyMOL (v3.1.6.1; Schrödinger, LLC). Surface electrostatic potentials were rendered using a − 10 to + 10 kT/e colour scale to visualize charge distribution across the protein surface.

## Electronic Supplementary Material

Below is the link to the electronic supplementary material.

Supplementary Material 1

Supplementary Material 2

## Data Availability

The authors declare that the main data supporting the findings of this study are available within the article. The sequence alignment on which the phylogenetic tree shown in Figure 3 is available in the Supplementary Material.
