# Supplementary material for: Evolutionary Prevalence of the Electrostatic Switch Mechanism in P-type ATPases
Source: J Membr Biol. 2026 May 26;259(1):16. doi: 10.1007/s00232-026-00377-4 (PMC13212812; doi:10.1007/s00232-026-00377-4)
Supplement: Supplementary file 1 — Supplementary Material 1 [file 232_2026_377_MOESM1_ESM.docx]

Sliding Window Analysis (Table 1)

library(Biostrings)
library(stringr)

canonical_sequences <- readAAStringSet("Canonical_sequences.txt")

tail_info <- read.table("Terminal_tails.txt",
 header = TRUE, sep = ",",
 stringsAsFactors = FALSE)
tail_info$ID <- trimws(tail_info$ID)

window_size <- 30
min_rk_count <- 9
positive_residues <- c("K", "R")

find_polybasic_sliding_window <- function(seq, window_size, min_rk_count) {
 seq_length <- nchar(seq)
 cluster <- list()

 for (i in 1:(seq_length - window_size + 1)) {
 window <- substr(seq, i, i + window_size - 1)
 rk_count <- sum(str_count(window, "[RK]"))

 if (rk_count >= min_rk_count) {
 cluster <- append(cluster, list(list(
 start = i,
 end = i + window_size - 1,
 rk_count = rk_count,
 window_seq = window)))
 }
 }
 return(cluster)
}

output_results <- lapply(1:length(canonical_sequences), function(i) {
 protein_id <- names(canonical_sequences)[i]
 seq <- as.character(canonical_sequences[[i]])
 protein_length <- nchar(seq)

 tail_row <- tail_info[tail_info$ID == protein_id, ]
 if (nrow(tail_row) == 0) return(NULL)

 n_tail_end <- tail_row$N_TailEnd
 n_tail_start <- 1
 if (!is.na(n_tail_end)) {
 n_tail_seq <- substr(seq, n_tail_start, n_tail_end)
 n_clusters <- find_polybasic_sliding_window(n_tail_seq, window_size, min_rk_count)
 if (length(n_clusters) > 0) {
 n_clusters <- lapply(n_clusters, function(cluster) {
 cluster$abs_start <- cluster$start + n_tail_start - 1
 cluster$abs_end <- cluster$end + n_tail_start - 1
 cluster
 })
 }
 } else {
 n_clusters <- NULL
 }

 c_tail_start <- tail_row$C_TailStart
c_tail_end <- tail_row$C_TailEnd
if (!is.na(c_tail_start) && !is.na(c_tail_end)) {
 c_tail_length <- c_tail_end - c_tail_start + 1
 c_tail_seq <- substr(seq, c_tail_start, c_tail_end)
 c_clusters <- find_polybasic_sliding_window(c_tail_seq, window_size, min_rk_count)
 if (length(c_clusters) > 0) {
 c_clusters <- lapply(c_clusters, function(cluster) {
 cluster$abs_start <- cluster$start + c_tail_start - 1
 cluster$abs_end <- cluster$end + c_tail_start - 1
 cluster
 })
 }
} else {
 c_clusters <- NULL
}

 clusters <- c(n_clusters, c_clusters)

 result_str <- paste0("Sequence: ", protein_id,
 "\nLength: ", protein_length, " amino acids\n")

 if (length(clusters) > 0) {
 result_str <- paste0(result_str, "Polybasic regions (≥9 R/K in 30aa):\n")
 cluster_info <- sapply(clusters, function(cluster) {
 nearby_seq <- substr(seq,
 max(1, cluster$abs_start - 5),
 min(protein_length, cluster$abs_end + 5))
 has_S <- grepl("S", nearby_seq)
 has_T <- grepl("T", nearby_seq)
 has_Y <- grepl("Y", nearby_seq)

 phospho_note <- if (has_S && has_T && has_Y) "S, T and Y nearby" else
 if (has_S && has_T) "S and T nearby" else
 if (has_S && has_Y) "S and Y nearby" else
 if (has_T && has_Y) "T and Y nearby" else
 if (has_S) "S nearby" else
 if (has_T) "T nearby" else
 if (has_Y) "Y nearby" else "No S, T or Y nearby"

 paste0(" Position ", cluster$abs_start, "–", cluster$abs_end,
 " | RK count: ", cluster$rk_count,
 " | Window: ", cluster$window_seq,
 " | ", phospho_note)
 })
 result_str <- paste(result_str, paste(cluster_info, collapse="\n"), "\n")
 } else {
 result_str <- paste0(result_str, "Polybasic regions:\n None\n")
 }

 return(result_str)
})

final_output <- paste(unlist(output_results), collapse="\n")

cat(final_output)
